# Chemical and Functional Properties of Chia Seed (*Salvia hispanica* L.) Gum

**DOI:** 10.1155/2014/241053

**Published:** 2014-03-23

**Authors:** Maira Rubi Segura-Campos, Norma Ciau-Solís, Gabriel Rosado-Rubio, Luis Chel-Guerrero, David Betancur-Ancona

**Affiliations:** Facultad de Ingeniería Química, Universidad Autónoma de Yucatán, Periférico Nte. Km. 33.5, Tablaje Catastral 13615, Col. Chuburná de Hidalgo Inn, 97203 Mérida, YUC, Mexico

## Abstract

Chia (*Salvia hispanica* L.) constitutes a potential alternative raw material and ingredient in food industry applications due to its dietary fiber content. Gum can be extracted from its dietary fiber fractions for use as an additive to control viscosity, stability, texture, and consistency in food systems. The gum extracted from chia seeds was characterized to determine their quality and potential as functional food additives. The extracted chia gum contained 26.2% fat and a portion was submitted to fat extraction, producing two fractions: gum with fat (FCG) and gum partly defatted (PDCG). Proximal composition and physicochemical characterization showed these fractions to be different (*P* < 0.05). The PDCG had higher protein, ash, and carbohydrates content than the FCG, in addition to higher water-holding (110.5 g water/g fiber) and water-binding capacities (0.84 g water/g fiber). The FCG had greater oil-holding capacity (25.7 g oil/g fiber) and water absorption capacity (44 g water/g fiber). In dispersion trials, the gums exhibited a non-Newtonian fluid behavior, specifically shear thinning or pseudoplastic type. PDCG had more viscosity than FCG. Chia seed is an excellent natural source of gum with good physicochemical and functional qualities, and is very promising for use in food industry.

## 1. Introduction

The Chia (*Salvia hispanica*) seed was used as an offering to the Aztec gods, and, because of its religious use, it essentially disappeared for 500 years. This is an annual herbaceous plant belonging to the Lamiaceae or Labiatae family. In pre-Columbian times, it was one of the basic foods of several Central American civilizations, less important than corn and beans, but more important than amaranth [1]. Seeds are consumed in México, Argentina, and the southwestern United States. The chemical composition reports contents of protein (15–25%), fats (30–33%), carbohydrates (26–41%), dietary fiber (18–30%), and ash (4-5%). It also contains a high amount of vitamins, minerals, and antioxidants [2].

Chia seeds have been investigated and recommended due to their high levels of proteins, antioxidants, dietary fiber, vitamins, and minerals but particularly due to their oil content with the highest proportion of *α*-linolenic acid (*ω*-3) compared to other natural sources known to date [3]. Chia seeds contain up to 39% of oil, which has the highest known content of *α*-linolenic acid, up to 68% [4].

Chia seed gum has the potential for industrial use because of its slimy properties, evident even at very low concentration, and because the plant, native to America, grows well in semiarid regions that have few practical plants. Chia gum begins to emerge from seeds as soon as they are placed in water. The gum appears to be contained in the seed coat or the adjacent layer. The exudate is either partially cross-linked or is bound to the seed surface, since it is not easily separated from the seed. Separation can be accomplished by strong stirring, preferably in the presence of sand to aid in dislodgment or cleavage of insolubilizing bonds. For research purposes, gum has been removed by extraction of seeds with a 6 M urea solution. Chia gum is composed of *β*-D-xylopyranosyl, *α*-D-glucopyranosyl, and 4-O-methyl-*α*-D-glucopyranosyluronic acid unit in the ratio 2 : 1 : 1. The polysaccharide seems to consist of a repeating unit. Extracted gum has a slimy, mucilaginous character at very low concentrations, giving it wide potential use in a variety of industrial applications, especially in certain foods and food preparations [5].

The objective of the present study was to determine the physicochemical properties of fatted and defatted gums from chia (*Salvia hispanica*) seeds.

## 2. Materials and Methods 

### 2.1. Materials

Chia (*S. hispanica* L.) seeds were obtained in the Yucatan State of Mexico. Reagents were of analytical grade and purchased from J. T. Baker (Phillipsburg, NJ, USA), Sigma (Sigma Chemical Co., St. Louis, MO, USA), Merck (Darmstadt, Germany), and Bio-Rad (Bio-Rad Laboratories, Inc. Hercules, CA, USA).

### 2.2. Fatted (FCG) and Partly Defatted (PDCG) Chia Gum

Seeds of chia were submitted to gum extraction with water at a 1 : 20 ratio (w/v) for 30 min and at a 50°C temperature. After that, the suspension was milled in a mixer and then it was boiled again at 50°C under stirring for 15 min. The crude mixture, containing water, gum, and seeds, was centrifuged at 9460 ×g at 15°C for 3 h. The recovered gum (FCG) was dried at −40°C for 24 hours and milled. One portion of the recovered gum was partly defatted (PDCG) in a Soxhlet.

### 2.3. Chemical Characterization of Fatted and Defatted Chia Gum

Standard AOAC [6] procedures were used to determine nitrogen (method 954.01), fat (method 920.39), ash (method 925.09), crude fiber (method 962.09), and moisture (method 925.09) contents in the fatted and defatted chia gums.

### 2.4. Functional Properties of Fatted and Defatted Chia Gum

#### 2.4.1. Water Absorption Capacity (WA_b_C)

This property was determined according to AACC method 88-04 [7]. Approximate water absorption capacity was first determined by weighing out 0.1 g (d.b.) of sample, adding water until saturation (approximately 5 mL), and centrifuging at 2000 ×g for 10 min in a Beckman GS-15R centrifuge. Excess water was discarded and the residue was weighed. Approximate water absorption capacity was calculated by dividing the increase in sample weight (g) by the quantity of water needed to complete original sample weight to 15 g. Water absorption capacity (WA_b_C) was then determined by placing samples in four tubes, adding different quantities of water to bracket the measurement (1.5 and 0.5 mL water above original weight and 1.5 and 0.5 mL water below; one in each tube), agitating vigorously in a vortex for 2 min, and centrifuging at 2000 ×g for 10 min in a Beckman GS-15R centrifuge. The supernatant was discarded and the residue was weighed. Average water absorbed was calculated and the WA_b_C was calculated, expressed as g water absorbed per g of sample.

#### 2.4.2. Water Adsorption Capacity (WA_d_C)

This property was determined according to Chen et al. [8]. Briefly, 0.1 g (d.b.) of sample was placed in an equilibrium microenvironment at 98% relative humidity, generated by placing 20 mL of saturated potassium sulfate saline solution in tightly sealed glass flasks and placing these in desiccators at 25°C. The sample was left in the microenvironment until reaching constant weight (72 h). Water adsorption capacity was expressed as g of water per g of sample.

#### 2.4.3. Water-Holding (WHC) and Oil-Holding Capacity (OHC)

Both capacities were determined following Chau et al. [9]. Briefly, 0.1 g (d.b.) of sample was weighed and then stirred into 20 mL of distilled water or corn oil (Mazola, CPI International) for one minute. These fibrous suspensions were then centrifuged at 2200 ×g for 30 min and the supernatant volume was measured. Water-holding capacity was expressed as g of water held per g of sample, and oil-holding capacity was expressed as g of oil held per g of gum. Corn oil density was 0.92 g/mL.

#### 2.4.4. Viscosity

Apparent viscosity was evaluated using an adaptation of the Li and Chang [10] method, using a Brookfield viscometer model DV-II (Brookfield Engineering Lab., Stoughton, MA) with spindle 27 (for small samples) and share rate range from 2.5 to 100 rpm at 25°C. Equilibrium time between measurements was 30 seconds. The samples were dispersed in water to 0.5, 1.0, 1.5, 2.0, and 2.5% (w/v, db). The results were expressed in Pa.s and data was fixed to an Ostwald-de Waele model to determine the consistency index (*k*) and flow behavior index (*n*).

### 2.5. Statistical Analysis

All experiments were carried out in triplicates. Data obtained were subjected to analysis of variance (ANOVA). Differences among means were determined using the Duncan multiple range test [11].

## 3. Results and Discussion

### 3.1. Chemical Characterization of Fatted and Partly Defatted Chia Gums

Chia gum's yield was 10.9%. This percentage was lower than reported by Sciarini et al. [12] in* Gleditsia triacanthos* seeds (11.9–34.16%). However, the chia gum's yield was higher than reported by Oomah et al. [13] in flaxseed (3.6–8%).

The proximal composition (Table 1) showed that PDCG registered a higher content of protein, ash, and NFE than FCG. The fiber content of FCG (28.96%) was similar to that reported by Vazquez-Ovando et al. [14] in a fiber-rich fraction of chia seeds (29.56%). The moisture content of both gums was similar to that reported by Kader et al. [15] in* Acacia glomerosa* (9.09%) but lower than reported in guar gum (10.36%), xanthan gum (11.08%), and* Gleditsia triacanthos* (14.08%) [12]. With respect to the NFE content, both gums registered lower values than reported by Vazquez-Ovando et al. [14] in a fiber-rich fraction of chia seeds (34.52%). However, the ash content of FCG and PDCG was higher than that reported by Kader et al. [15] in Arabic gum (*Acacia Senegal*, 3.6%) although lower than reported by Sciarini et al. [12] in xanthan gum (9.35%). The protein content of both gums was higher than registered in corn gum (5.1%) and mesquite gum (5.8%), this last one with important emulsifying properties attributed to its protein content according to Bosquez [16]. In this respect, López et al. [17] establish that hydrocolloids rich in protein, such as gelatin, Arabic gum, and mesquite, are good stabilizers because they have sufficient hydrophobic groups to act as bonding points as well as hydrophilic groups that reduce surface tension in a liquid-liquid or liquid-gas interface. On the other hand, Yadav et al. [18] establish that the lipid content in the gums may also play an important role in stabilization of oil-water emulsions. However, Bosquez [16] established that carbohydrates avoid flocculation and coalescence of oil droplets to extend in the aqueous solution. These findings suggest that FCG and PDCG could act as good emulsifiers and stabilizers in the food industry.

### 3.2. Functional Properties of Fatted and Partly Defatted Chia Gums

**Figure 1:**
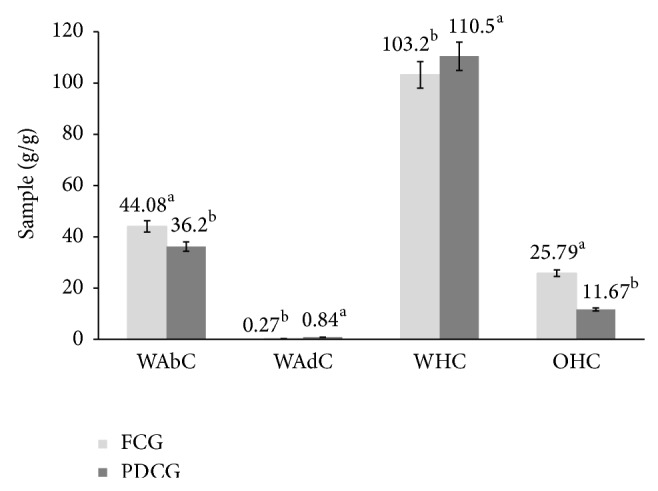
Functional properties of fatted and partly defatted chia gum. Water absorption capacity (WAbC), water adsorption capacity (WAdC), water holding capacity (WHC), and oil holding capacity (OHC).  ^a-b^Different superscript letters in the same property indicate statistical difference (*P* < 0.05).

Figure 1 shows the functional properties of fatted and defatted chia gum. Water absorption capacity is indicative of a structure's aptitude to spontaneously absorb water when placed in contact with a constantly moist surface or when immersed in water. Water adsorption capacity is the ability of a structure to spontaneously adsorb water when exposed to an atmosphere of constant relative humidity [19]. WAbC was higher in FCG (44.08 g/g of sample) than PDCG (36.2 g/g of sample). The high values of WAbC obtained here could be due to the proteins present in the gums, which would have a large number of exposed hydrophilic sites interacting with water [20]. The WAbC of FCG and PDCG was higher than reported by Vázquez-Ovando et al. [21] in a fiber-rich fraction of chia seeds (11.73 g/g of sample), who establish that fiber content is an important factor in the increment of this property for its capacity to form gels and to hold water; this justifies the higher value of WAbC in FCG. On the other hand, PDCG (0.84 g/g of sample) registered a higher value of WAdC than FCG (0.27 g/g of sample). The WAdC of DCG was also higher than registered by Vázquez-Ovando et al. [21] in a fiber-rich fraction of chia seeds (0.3 g/g of sample), similar to that reported in carrots (0.82 g/g of sample) but lower than the value registered in beet bagasse (1.58 g/g of sample).

PDCG showed a higher value of WHC than FCG. However, WHC of both gums was higher than that reported by Vázquez-Ovando et al. [21] and Baquero and Bermúdez [22] in a fiber-rich fraction of chia seeds (15.41 g/g of fiber) and passion fruit peel (8.7 g/g of fiber), respectively. A similar behavior was observed with orange waste (7.65–8.23 g/g of fiber) [23]. Soluble fiber and the denaturalized proteins may have increased the WHC of both gums, thus enhancing the swelling ability [24], an important function of proteins in preparation of viscous foods such as soups, gravies, dough, and baked products. On the other hand, FCG showed a higher OHC than PDCG, which might be related to its higher value of fat. However, both gums registered higher OHC values than those registered in guar and xanthan gum (4–6 g oil/g fiber) although similar to that reported in Arabic gum (8-9 g oil/g fiber). This functional property has been attributed to the physical entrapment of oil for molecules such as lipids and proteins. For the above mentioned, the OHC registered in chia gums could be due to protein and fat contents as well as factors as particle size and the absence of hemicellulose. Chia gum seems to possess an adequate fat absorption capacity, allowing it to play an important role in food processing, since fat acts on flavor retainers and increases the mouth feel of foods.

**Figure 2:**
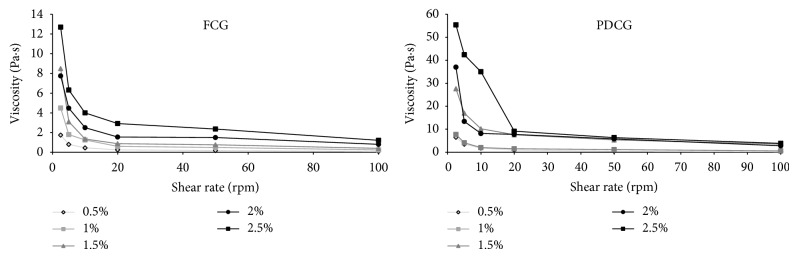
Viscosity (Pa·s) profiles of FCG and PDCG dispersions at different concentrations.

Figure 2 shows the viscosity profiles of FCG and PDCG dispersions. Both gums showed a non-Newtonian behavior where viscosity presented a relation directly proportional to the concentration and inversely proportional to the shear rate. The maximum viscosity reached between both gums was registered by PDCG (55.4 Pa·s) at 2.5%. In general, PDCG registered a higher viscosity profile than FCG suggesting that the fat content was the principal factor that generated this behavior. At this respect, Altunakar et al. [25] report that gums with higher oil absorption as Arabic gum show less viscosity (2.34 Pa·s at 3.8%). According to Table 2, the rheological behavior of the FCG and PDCG dispersions was a shear thinning or pseudoplastic type due to registered values of *n* < 1. The results suggest the use at low concentrations of DCG in products as yoghurts, sauces, toppings, and pastries among others that require high viscosity, whilst FCG could be used in sauces, mayonnaises, and meat products as emulsifying and stabilizer.

## 4. Conclusions

The results obtained here show that chia gums present interesting physicochemical properties for the food industry. The partly defatted chia gum showed a very good ability to water holding (110.5 g/g); however, their ability of oil holding (11.67 g/g) and water absorption (36.26 g/g) was minor compared to the fatted chia gum, which provided a greater retention of oil holding (25.79 g/g) and water absorption (44.08 g/g). Rheological behavior of gums was shear thinning or pseudoplastic type. From a functional point of view, chia gum also is an important food ingredient due its emulsifier and stabilizer potentials.

## Figures and Tables

**Table 1:** Proximal composition of fatted and partly defatted chia gum.

Component	% d.b.	
	FCG	PDCG
Moisture	(9.32)^a^	(8.95)^b^
Protein	25.07^a^	33.26^b^
Fiber	28.96^a^	18.99^b^
Fat	26.24^a^	10.90^b^
Ash	5.48^a^	8.28^b^
NFE	14.25^a^	28.41^b^

^a-b^Different letters in the same row indicate statistical difference *P* < 0.05.

**Table 2:** Consistency and behavior index of fatted and partly defatted chia gum.

Concentration (%)	Consistency index (*k*)		Behavior index (*n*)	
	FCG	PDCG	FCG	PDCG
0.5	0.52	1.87	0.2263	0.0658
1	1.33	2.70	0.2266	0.3472
1.5	2.07	12.06	0.2676	0.4624
2	3.05	11.75	0.4268	0.4114
2.5	4.87	23.39	0.4291	0.2184
